# The SENSE Study (Sleep and Education: learning New Skills Early): a community cognitive-behavioural therapy and mindfulness-based sleep intervention to prevent depression and improve cardiac health in adolescence

**DOI:** 10.1186/s40359-015-0096-x

**Published:** 2015-11-04

**Authors:** Joanna M. Waloszek, Orli Schwartz, Julian G. Simmons, Matthew Blake, Laura Blake, Greg Murray, Monika Raniti, Ronald E. Dahl, Neil O’Brien-Simpson, Paul Dudgeon, John Trinder, Nicholas B. Allen

**Affiliations:** Melbourne School of Psychological Sciences, The University of Melbourne, Parkville, VIC 3010 Australia; Psychological Sciences and Statistics, Swinburne University of Technology, Hawthorn, VIC 3122 Australia; School of Public Health, University of California, Berkeley, CA 94720-7369 USA; Department of Psychology, University of Oregon, Eugene, OR 97403-1227 USA; Melbourne Dental School, Oral Health CRC, The University of Melbourne, Parkville, VIC 3010 Australia

**Keywords:** Sleep, Adolescence, Intervention, Cardiovascular, Mindfulness, Cognitive, Anxiety, Depression, Prevention, Behavior

## Abstract

**Background:**

Sleep problems are a major risk factor for the emergence of depression in adolescence. The aim of this study was to test whether an intervention for improving sleep habits could prevent the emergence of depression, and improve well-being and cardiovascular indices amongst at-risk adolescents.

**Methods/Design:**

A longitudinal randomised controlled trial (RCT) is being conducted across Victorian Secondary Schools in Melbourne, Australia. Adolescents (aged 12–17 years) were defined as at-risk for depression if they reported high levels of anxiety and sleep problems on in-school screening questionnaires and had no prior history of depression (assessed by clinical diagnostic interview). Eligible participants were randomised into either a sleep improvement intervention (based on cognitive behavioral and mindfulness principles) or an active control condition teaching study skills. Both programs consisted of seven 90 minute-long sessions over seven weeks. All participants were required to complete a battery of mood and sleep questionnaires, seven-days of actigraphy, and sleep diary entry at pre- and post-intervention. Participants also completed a cardiovascular assessment and two days of saliva collection at pre-intervention. Participants will repeat all assessments at two-year follow up (ongoing).

**Discussion:**

This will be the first efficacy trial of a selective group-based sleep intervention for the prevention of depression in an adolescent community sample. If effective, the program could be disseminated in schools and greatly improve health outcomes for anxious adolescents.

**Trial registration:**

Australian New Zealand Clinical Trials Registry ACTRN12612001177842. Date of Registration: 06-Nov-2012.

**Electronic supplementary material:**

The online version of this article (doi:10.1186/s40359-015-0096-x) contains supplementary material, which is available to authorized users.

## Background

Adolescent depression is both common and harmful, with an estimated 15–20 % of adolescents experiencing clinical depression [1]. Depression is strongly associated with disturbed sleep [2], a relationship that is particularly marked in adolescence [3], and there is accumulating evidence that disturbed sleep can play a *precipitating* role in the onset of depression and other problems during adolescence [4]. The likelihood that sleep disturbance plays a critical etiological role in adolescent depression suggests that sleep improvement might decrease risk for the development of depression. Moreover, improved sleep may benefit other aspects of health, including cardiovascular health [5]. There is a complex relationship between depression, sleep and cardiovascular disease (CVD) across the lifespan [6], suggesting that early intervention for sleep may impact on a mechanism jointly associated with risk for CVD and depression. The potential public health benefits of effective early intervention for sleep problems are therefore substantial, and a treatment trial is warranted.

### Depression, anxiety and sleep disturbance in adolescence

In adolescence, there is a significant rise in depression incidence [1] and increased risk for a deterioration in the quantity and/or quality of sleep [7]. Factors that appear to contribute to adolescent vulnerabilities to sleep problems include maturational changes in both the homeostatic and circadian regulation of sleep [8, 9], less parental control over bedtime, as well as the development of cultural and social interests and obligations such as homework, hobbies and use of electronic media in the evening that interfere with bedtime. Importantly, these factors often appear to interact with each other contributing to late-night and erratic sleep onset times, and these interact with (relatively early) school starting times to reduce sleep duration [10]. Research has also shown that anxious youths may be at particular risk for sleeping difficulties [11–15]. Importantly, anxiety often precedes the emergence of depressive disorders and the onset of insomnia, whereas episodes of depression follow bouts of insomnia [16], suggesting that sleep disturbance might serve as a mediating link between anxiety and depression.

### Disturbed sleep and depression

Sleep problems are cross-sectionally associated with adolescent depression [17], and recent longitudinal studies have demonstrated that sleep problems are also prospectively associated with depression in adolescents [18–21]. There is also emerging evidence that manipulations of adolescents’ sleep can modify psychological factors, including depressive symptoms [22, 23]. These findings suggest that targeting sleep problems in early-to-mid adolescents who have high levels of anxiety and concomitant sleep problems may constitute an effective targeted prevention approach to depression in this age group.

### Depression, sleep disturbance and heart disease

There is strong evidence linking poor sleep and heart disease [24, 25], and in young people, poor sleep quality and sleep disorders have been associated with risk factors for later cardiac disease [26–31]. Furthermore, treatment of youth sleep disorders has been associated with a reduction in cardiovascular disturbances [29].

The relationship between depressive disorders and cardiac disease is also well-established [32], although the mechanisms underlying the association are not yet well understood [33]. Depression is a significant predictor of the onset of coronary artery disease [34] as well as of cardiac mortality in patients with coronary heart disease [33, 35]. Although case level CVD in those vulnerable to depression will typically emerge in later life, there are now strong indicators that, during adolescence, depression is associated with cardiovascular abnormalities that may be early indicators of compromised cardiovascular health [36–41]. Improving the quality of sleep in adolescents who are at risk for depression may therefore present a viable early intervention that improves both cardiovascular and mental health. To date no such study has been undertaken.

### Mechanisms underling the association between depression and CVD

A number of mechanisms have been identified as potential links between depression and CVD, and may also constitute early indicators of the development of CVD. These include disturbance in autonomic cardiac control, vascular endothelial dysfunction in coronary arteries, and immune system activation (see [32], for a recent comprehensive review). Many markers of systemic inflammation that have been found to be elevated in depressed persons, such as IL-6, tumor necrosis factor-α (TNF-α), and C-reactive protein (CRP) [42] have also been shown to be predictive of CVD [43]. Although there is no definitive agreement as to which of these mechanisms might be most critical to the link between CVD and depression, they each enjoy preliminary support. Importantly, no studies have comprehensively characterised these aspects of cardiac functioning in young people at risk for depression; nor has any study investigated whether a sleep intervention might modify such risk factors. The proposed study will address both of these critical issues.

### Study aims and main objectives

The aim of the SENSE (Sleep and Education: learning New Skills Early) Study is to determine the preventative effect of a sleep improvement intervention on the emergence of depression in an at-risk adolescent population. The project also provides the opportunity to test a potential nexus between sleep, depression and CVD, and to measure the intervention’s effects on early indices of risk for CVD. Specifically it is hypothesized that, relative to an active study skills control intervention (Study SENSE):

1. A brief sleep intervention (Sleep SENSE) will improve both subjective and objective indices of sleep quality in a sample of at risk adolescents, and that this improvement will persist at a two-year follow-up.
2. The sleep intervention will decrease reports of anxiety and mood symptoms immediately after the intervention and prevent the onset of case-level depression over a two-year follow-up period in a sample of at risk adolescents.
3. The sleep intervention will improve indices of cardiovascular health at two-year follow-up. In particular, levels of IL-1α, IL-1β, TNFα, IL-6, and C-reactive protein will decrease and endothelial function will improve.
4. Benefits to both mental and cardiovascular health will be mediated by the measured improvements in sleep that result from the sleep intervention.

## Methods

### Design

The project is a longitudinal parallel randomised controlled trial (RCT) in which the experimental group took part in a CBT/mindfulness-based sleep intervention (Sleep SENSE) and the active control group took part in a study skills educational program (Study SENSE). The control intervention was chosen to have strong face validity as a well-being and/or performance enhancing intervention for adolescents, and to entail similar levels of effort and engagement with interventionists as did the Sleep SENSE intervention. Participants were recruited via a school-based screening to identify students from the general community with high levels of anxiety and sleeping difficulties. Participants underwent assessments of sleep and psychopathology before and immediately after the intervention phase, and will again at the two-year follow-up. Cardiovascular health was assessed before the intervention and will be re-assessed at the two-year follow-up. As adolescent sleep is strongly affected by school schedules [44], the intervention and sleep assessments were timetabled during school term time.

### Ethics, consent and permissions

Participants were recruited from secondary schools in the Melbourne Metropolitan Area, Australia. Pre/post-intervention data collection was conducted in the Melbourne School of Psychological Sciences at the University of Melbourne, Australia. Interventions were also held at the University, except for one group that was held at the participants’ school. The study and all procedures, including data management and participant confidentiality, were approved by the University of Melbourne Human Research Ethics Committee (HREC#1237312), the Department of Education and Early Childhood Development (DEECD) (2012_001659), and the Catholic Education Office Melbourne (CEOM) (GE12/000091819), and complied with National Health and Medical Research Council guidelines. All participants and their guardians gave written informed consent before participating in the study. The SENSE Study is registered in the Australia and New Zealand Clinical Trials Registry (ACTRN12612001177842) and funding for the project was received through the Australian National Health and Medical Research Council (APP1027076) (Additional files 1 and 2).

### Procedure

**Fig. 1:**
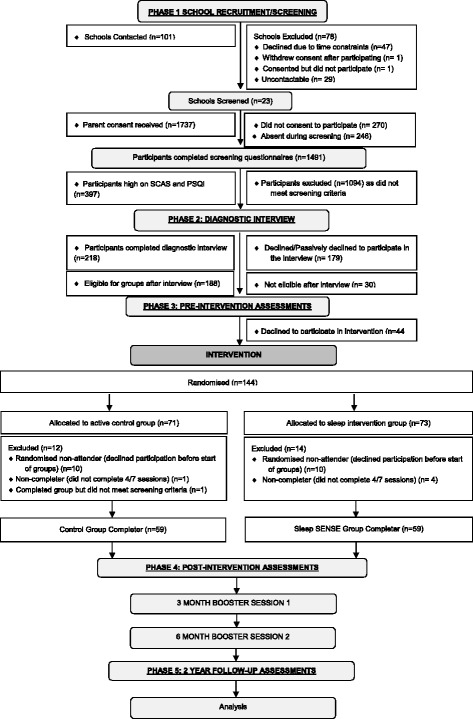
Flowchart of participation in each phase of the SENSE Study to date

The SENSE Study has five data collection ‘Phases’ in addition to the Intervention itself. Details of the phases, recruitment process and participant numbers at each phase can be found in Fig. 1. Phases 1–4 (screening-post-intervention assessments) have been completed and Phase 5 (2-year follow up assessments) is ongoing. Participants were reimbursed for their time and travel expenses with a department store voucher for each assessment during Phases 2–5.

### Participant recruitment

Participants were recruited using a two-stage procedure, consisting of an in-school screening followed by a diagnostic interview for those meeting screening criteria, to identify students with high levels of anxiety and sleeping difficulties but without a history of depressive disorder. Selected adolescents were then invited to take part in the trial (Phases 2–5 of the study).

Schools were selected and approached based on geographical proximity (within 35 km) to the University of Melbourne, where assessments would take place. Schools were contacted via letters or emails describing the study in detail. Schools were offered information booklets and tailored presentations on adolescent wellbeing to increase interest in the study. Schools who did not wish to participate in the study indicated they did not have enough time due to a full curriculum, were already participating in other research studies (i.e., decline) or the school coordinator was not contactable (i.e., passive decline).

All students in Years 7–10 were invited to participate in the study; however the school coordinator determined which classes would participate, based on student time commitments, relevance to the teaching curriculum and staff limitations. Students who provided written parental consent (hardcopy or online) were asked to attend the screening assessment session in a designated teacher-supervised classroom during school time. Researchers explained the study via standardised instructions and students who wished to continue were asked to complete the screening questionnaire pack (see Table 1 for details of measures). The questionnaires took approximately 20 min to complete.

**Table 1:** Summary of measures administered at each data collection point

Category	Measures	Screening	Pre-Intervention	Post-Intervention	Booster 1 & 2	Two-year Follow-up
Psychopathology and Global Functioning	SCAS	x	x	x	x	x
	CES-D	x	x	x	x	x
	K-SADS-PL		x	x		x
	YRBS-Sub		x	x		x
	CBCL-Ext		x	x		x
	CGAS		x	x		x
	LIFE-I					x
Global Cognitive Style	PSWQ-C		x	x	x	x
	RSQ-Rumination		x	x	x	x
	GSES		x	x	x	x
Sleep-Specific Cognitive Style	SBS		x	x	x	x
	DBAS-16		x	x	x	x
	PAS		x	x	x	x
Sleep	PSQI	x	x	x	x	x
	rMEQ	x				
	PDSS		x	x	x	x
	Sleep Diary		x	x		x
	Actigraphy		x	x		x
Cardiovascular Health	Saliva samples		x			x
	Blood Pressure		x			x
	Heart rate		x			x
	Endothelial dysfunction		x			x
	Medical history questionnaire		x			x

*CBCL-Ext* Child Behaviour Checklist – Externalizing scale, *CES-D* Center for Epidemiologic Studies – Depression, *CGAS* Children’s Global Assessment of Functioning, *DBAS-16* Dysfunctional Beliefs about Sleep – 16 item version, *GSES* General Self-Efficacy Scale, *K-SADS* Schedule for Affective Disorders and Schizophrenia – Children’s version, *LIFE-I* Longitudinal Interval Follow-up Evaluation Interview (Student work, Interpersonal Relations with Family, Depressive Disorders), *rMEQ* reduced Morningness-Eveningness Questionnaire, *PAS* Pre-sleep Arousal Scale, *PDSS* Pediatric Daytime Sleepiness Scale, *PSQI* Pittsburg Sleep Quality Index, *PSWQ-C* Penn State Worry Questionnaire - Children, *RSQ-Rumination* Response Scale Questionnaire – Rumination subscale, *SBS* Sleep Beliefs Scale, *SCAS* Spence Children’s Anxiety Scale, *YRBS-Sub* Youth Risk Behavior Survey – Subscale use questions

### Inclusion & exclusion criteria

Participants were required to have an adequate comprehension of English written and spoken language to participate in the study. Participants whose ratings on the screening questionnaire indicated high anxiety on the Spence Children’s Anxiety Scale ([SCAS], i.e. a Total Score >32 and >38 for males and females respectively) [45], as well as the likely presence of sleep problems as identified by the Pittsburgh Sleep Quality Index ([PSQI], i.e. a Global Score ≥ 5) [25], were invited to take part in a face-to-face diagnostic interview based on DSM-IV-TR criteria (the Kiddie Schedule for Affective Disorders and Schizophrenia for School-Age Children-Present and Lifetime Version [K-SADS-PL]) with trained interviewers. The interview was completed in the participant’s home or at the University of Melbourne. Participants who scored above the cut-off in the SCAS and PSQI in the screening assessment, and who had never met criteria for Major Depressive Disorder, as assessed using the K-SADS-PL, were invited to participate in the pre-intervention baseline assessments and group sessions.

### Baseline data collection

Participants who met inclusion criteria after the diagnostic interview were asked to complete a number of assessments prior to the group sessions. A week prior to the group session, participants were sent a ‘Welcome Pack’ which included mood and sleep questionnaires, a sleep diary, an Actiwatch and a saliva collection kit. Participants were asked to wear the Actiwatch and complete the sleep diary for the seven days prior to the commencement of the groups, as well as collect six saliva samples over two days, complete the questionnaires and return all materials to researchers at the first group session. In cases where participants decided not to attend the group sessions, a return post parcel was sent to participants to retrieve any data.

Participants were also invited to participate in a cardiovascular assessment conducted at the University of Melbourne Sleep Laboratory. In order to control for circadian variation in cardiovascular variables, the assessment was conducted between the hours of 2:30 pm and 6 pm. Participants were asked to reschedule if sick and to refrain from consuming any food or drink other than water for at least three hours prior to the assessment, and from taking any medication in the 24 h prior to the assessment. Upon arrival, participants were asked questions about their demographic and medical history, and their weight, height, percentage fat, waist and hip circumference were measured. All cardiovascular measurements were conducted in a seated position in a quiet room with dim lighting and constant temperature of 22° Celsius. After 5 min of quiet rest, three automatic measurements of brachial blood pressure and heart rate were taken, with a two-minute rest between each measurement. Following these measures, continuous beat-to-beat blood pressure and heart rate were monitored at rest for 15 min. Finally, endothelial function following brachial occlusion was also tested using standard procedures [46]. The cardiovascular assessment took approximately 1.5 h.

### Randomisation and blinding

After all Phase 1 assessments had been conducted (i.e., the interview and questionnaires, sleep and cardiovascular assessments), eligible participants who consented to participate in the intervention stage of the trial were randomly allocated to receive either the sleep intervention (Sleep SENSE) or the active control group (Study SENSE). A blinded statistician randomised the eligible participants stratified by gender, age and type of anxiety disorder at baseline using a minimisation method available in the MINIM program (SJW E, SJ D, P R. MINIM: minimisation programme for allocating patients to treatment in clinical trials. Unpublished document Department of Clinical Epidemiology, The London Hospital Medical College, London. 1990.) Participants and their guardians were made aware of the content of the group sessions (i.e., study skills vs. sleep skills) but not of the status of each group (i.e., intervention versus control) or the expected outcome of the study. Researchers conducting the post-intervention interviews were also blinded to which group participants had completed.

### Group sessions

As adolescent sleep is strongly affected by school schedules, the intervention and pre-/post-intervention sleep assessments were timetabled during school term time. Participants who did not attend at least four sessions were counted as ‘non-completers’.

### Intervention group sessions

Sleep SENSE builds on the work of Dahl, Bootzin and Harvey [47–51] and was successfully piloted among Australian adolescent girls, demonstrating preliminary effects on both subjective and objective measures of sleep and self-reported anxiety [48]. Sleep SENSE is a multi-component group program designed to improve sleep by addressing barriers to sleep across the sleep-wake cycle. It aims to improve sleep quality in the short term and, via sustained behavior change, the long-term, and is tailored to address the unique developmental challenges and opportunities of adolescence. Like evidence-based adult treatments for insomnia, the intervention is cognitive-behavioral in approach, incorporating sleep hygiene, stimulus control, cognitive-behavior therapy and mindfulness-based therapy, and has a specific focus on identifying barriers to change via motivational interviewing. It involves 7 weekly 90-minute group sessions supported by a range of psycho-educational materials and at-home tasks. Psychologists or psychologists in training facilitated interventions sessions. A summary of the content covered in each session is displayed in Table 2. Parents/care-givers were given information sheets about the material covered in each session to ensure the adolescent’s sleep improvement goals were integrated and supported by the family.

**Table 2:** Session outline of the sleep intervention and active control groups

Session	Sleep Intervention Group (Sleep) SENSE Content	Control Group (Study SENSE) Content
1	Introduction: education about sleep; identifying personal sleep goals; developing motivation to change	Introduction: why good study skills and habits are important for academic success.
2	Overcoming challenges to sleep: discuss good sleep hygiene and barriers to sleep, learn stimulus control strategies; introduce mindfulness and mindfulness of the breath practice.	Personal organization, time management and the home study environment.
3	Establishing a regular sleep schedule: learn about circadian rhythms and guidelines for keeping regular sleep schedule and limiting media use at bedtime; design a personal sleep plan; mindfulness of the breath practice.	Active listening, learning, and note-taking strategies.
4	Techniques for Managing Stress: learn about mindfulness, mindfulness qualities and their benefits for sleep; practice mindful attention, mindfulness of the breath and the body scan	Memory, memorization techniques, and different ways of learning.
5	Focusing on the Positive: learn about the cognitive-behavioural model; learn to identify and challenge unhelpful beliefs about sleep; practice savouring and switching and mindfulness of the breath	Test-taking, critical reading and essay writing strategies.
6	Managing worries: learn about the nature of worries and solvable versus unsolvable problems; strategies for managing worries during the day (problem solving, scheduled worry, worry box) and at night (mindfulness, savouring and switching); practice new mindfulness strategies (the 3-minute breathing space & 'letting go' techniques).	Public speaking and speech writing
7	Your sleep into the future: review of sleep goals and progress; program review; setback prevention; final mindfulness practice.	Review of Study SENSE program and problem solving strategies.

### Control group sessions

Study SENSE was administered by a trained education teacher and a co-facilitator for the same duration, and in the same format as the Sleep SENSE intervention. Components of the study skills group included writing persuasive essays, referencing, note taking and public speaking (see Table 2 for a summary of the content in each session).

### Post-intervention follow-up & booster sessions

Upon completion of the group sessions, all participants were re-administered the mood and sleep questionnaire pack first completed at pre-intervention, and were asked to wear an Actiwatch and complete a sleep diary for the following seven days. Assessment packs were distributed in session seven in both the Sleep and Study SENSE groups or it was sent to them at home if participants did not attend the session. Participants also completed another diagnostic interview, this time examining symptoms since the pre-intervention interview. The interview was conducted by trained researchers over the phone or face-to-face at the University of Melbourne and took an average of 45 min to complete. All researchers conducting post-intervention interviews were blinded to the group that the participants completed.

Following the group sessions, all participants were invited to attend two ‘booster’ sessions, held at the University of Melbourne at three and six months post-intervention. In the booster sessions, components of the groups were revised, any problems were discussed, and questions were answered. No new content was introduced. At the end of each booster session, participants were asked to complete the same mood and sleep questionnaire pack given to them at pre- and post-intervention. The questionnaire pack and booster session notes were sent to participants who did not attend the sessions with the option of sending back the completed pack.

### Two-year longitudinal follow-up

Participants will be contacted again two years after the completion of their group sessions. This longitudinal follow-up is ongoing. Participants will be asked to complete a ‘Follow-up Pack’ that will include the same contents as the pre-intervention ‘Welcome Pack’ (mood and sleep questionnaires, saliva collection kit, and an Actiwatch to wear and sleep diary to complete over seven days). Participants will also be asked to take part in a cardiovascular assessment and a clinical diagnostic interview that will explore symptoms experienced since the post-intervention interview. The *Longitudinal Interval Follow-up Evaluation Interview (LIFE-I)* [52] will also be included in the follow-up interview. All researchers conducting two-year follow-up interviews will be blinded to the group that the participants completed.

### Measures

#### Psychopathology measures

-  
*Spence Children’s Anxiety Scale (SCAS)* [53] - The SCAS has been shown to be an effective screening instrument for anxiety disorders in the targeted age group. The SCAS is a brief self-report test of anxiety symptoms broadly in line with the dimensions of anxiety disorder proposed by the DSM-IV. The scale assesses six domains of anxiety including generalized anxiety, panic/agoraphobia, social phobia, separation anxiety, obsessive-compulsive disorder and physical injury fears. The SCAS has normative data in the relevant age range and has been shown to have good internal consistency and temporal stability three months apart among 12–15 year olds [54, 55]. This study used the recommended total SCAS cut-offs of >32 for males and >38 for females [45].
-  
*Center for Epidemiologic Studies – Depression Scale (CES-D)* [56] - The CES-D is a reliable and well-validated 20-item self-report questionnaire measuring symptoms of depression during the past week [56]. The CES-D has also been validated as a reliable measure for the use in adolescents [57–59].
-  
*Kiddie Schedule of Affective Disorders and Schizophrenia Children's Version - Present and Lifetime Version* (K-SADS-PL) [60] – The KSADS-PL is a semi-structured diagnostic interview widely used for research of mood disorder in children and adolescents. It has been shown to be a reliable and valid measure of DSM-IV Axis 1 disorders in this population [61]. The following modules were administered: depression, mania, psychosis, panic disorder, social phobia, specific phobia/agoraphobia, generalised anxiety, obsessive-compulsive disorder, separation anxiety, and post-traumatic stress disorder. Interviews were conducted by trained interviewers and audio recorded. Regular clinical supervision was provided to all interviewers. Approximately 20 % of interviews were double-scored by another interviewer for inter-rater reliability.
-  The *Youth Risk Behavior Survey (YRBS)* [62] assesses health-risk behaviors in youths. Thirty-two items that assess tobacco, alcohol and other drug use were administered.
-  
*The Child Behavior Checklist, Youth Self Report version (CBCL-YSR)* [63] – The CBCL-YSR is a widely used instrument assessing internalizing and externalizing problem behaviors in young people aged 11 to 18 years. The present study administered the 45-item Externalizing subscale only.
-  
*Penn State Worry Questionnaire for Children (PSWQ-C) -* The PSWQ–C is a 14-item self-report questionnaire designed to examine the generality, excessiveness and uncontrollability of worry in children and adolescents. It has excellent internal consistency (*α* = .90) and temporal stability (*r* = .92) among 12- to 18-year-olds [64].
-  
*Rumination Responses Scale (RRS); subscale of the Response Styles Questionnaire (RSQ-R) -* The RRS is a widely used and well-validated self-report questionnaire that assesses the predisposition to focus on or ruminate on depressed mood. The RRS includes 22 items describing responses to mood that are self-focused, symptom focused and consequence-focused [65]. It has been shown to have adequate psychometric properties with an adolescent sample [66].
-  
*General Self-Efficacy Scale (GSES) -* The General Self-Efficacy Scale is a 10-item self-report questionnaire designed to assess a broad and stable sense of personal competence to deal effectively with a wide range of demanding or novel situations [67]. The GSES is widely used and has been shown to have high reliability, stability and construct validity [68].
- *Longitudinal Interval Follow-up Evaluation Interview (LIFE-I)* [52] The LIFE-I is a semi-structured interview used to assess the longitudinal course of participants’ psychiatric symptoms, mental health treatment and psychosocial functioning.

#### Sleep measures

##### Subjective measures

-  
*Sleep Diary* is a widely used sleep questionnaire that collects information on daily sleep onset, morning awakening, and sleep quality.
-  The *Pittsburgh Sleep Quality Index (PSQI) –* the PSQI is a validated self-rated questionnaire used to assess subjective sleep quality and disturbances and the impact of poor sleep on functioning [69]. Adolescent sleep schedules are known to shift dramatically across the week [70]. To explore this shift, the first four questions of the PSQI were altered to include a rating for sleep during the week (i.e., Monday-Friday) as well as a separate rating for weekend (i.e., Saturday-Sunday). This study used the cut-off of a total PSQI of 5 and above [25].
-  
*The reduced Morningness-Eveningness Questionnaire (rMEQ)* - The rMEQ was developed from the original Horne-Ostberg Morningness-Eveningness-Questionnaire [71]. It consists of 5 items from the original questionnaire (Items 1, 7, 10, 18, and 19), which determine individual chronotype on a single scale with minimum and maximum values from 4 to 25, where higher scores indicate a tendency towards morningness. The conventional classification for the scores are from 4–7 (Definitely-Evening), 8–11 (Moderately-Evening), 12–17 (Neither), 18–21 (Moderately-Morning) and 22–25 (Definitely-Morning) [72]. Although this measure has not been widely used for adolescents, it correlates highly (*r* = 0.90) with the Morningness-Eveningness Questionnaire [73] which has been validated [74] and used in adolescent samples [75].
-  
*Dysfunctional Belief and Attitudes about Sleep Scale – 16 (DBAS-16) -* The DBAS-16 is an abbreviated form of the original 30-item DBAS [76] and was designed to assess dysfunctional sleep-related cognitions. The factor structure of the brief form is similar to the original 30-item version, with four factors reflecting a) perceived consequences of insomnia, (b) worry/helplessness about insomnia, (c) sleep expectations, and (d) medication [77]. It has been shown to have good internal consistency (*α* = 0.77 for clinical and *α* = 0.79 for research samples) and temporal stability (*r* = 0.83), and correlates with other self-report measures of insomnia severity, anxiety and depression [77].
-  
*Pre-Sleep Arousal Scale (PAS) -* The PAS is a 16-item self-report questionnaire designed to measure cognitive arousal (items 9–16; e.g., “worry about falling asleep”) and somatic arousal (items 1-8; e.g., “cold feeling in your hands, feet or your body in general”) prior to sleep [78]. The PAS is commonly used in adults but has shown good internal consistency in much younger populations (*α* = 0.85; each subscale *α* = 0.75 [79]). PAS scores are able to differentiate clinical from community samples and correlate significantly with anxiety and sleep measures.
-  
*Paediatric Daytime Sleepiness Scale (PDSS) -* The PDSS is an 8-item self-report questionnaire designed to assess daytime sleepiness in children and adolescents [80].
-  
*Sleep Beliefs Scale (SBS) -* The SBS is an 20-item self-report questionnaire designed to assess general beliefs about sleep, including the influence of substances, diurnal behaviours and pre-sleep activities and thoughts on sleep [81]. The SBS is based on the Sleep Hygiene Awareness and Practice Scale (SHAPS) [82, 83], designed for use in clinical and non-clinical populations [84, 85]. The SBS has three factors: (1) sleep-incompatible behaviours, (2) sleep-wake cycle behaviours and (3) thoughts and attitudes about sleep [81]. The total and subscale scores have been shown to have acceptable internal consistency in non-clinical samples (total score *α* = 0.71, subscale *α* range = 0.47–.63) [81].

##### Objective measures

-  
*Actigraphy.* Objective sleep was assessed using Actiwatch-64, Actiwatch-L and Actiwatch 2 (Mini-Mitter Company, Sun River, OR, USA) wristwatch monitors of physical activity used to assess sleep-wake patterns in normal environment over extended periods of time. Actigraphy has been well validated and tolerated in adolescent populations [86].

#### Medical history measure

-  A medical history questionnaire used in previous studies [87] was administered in interview form, and includes items about chronic or current illnesses, family history of cardiovascular disease, substances consumed on the day, and measures of height, weight and waist circumference.

#### Cardiovascular measures

-  
*Blood Pressure:* Blood pressure was measured using a continuous finger blood pressure device (Portapres, Model 2). This apparatus provides continuous assessment of BP using finger cuffs. It also provides an automated height adjustment feature. Maximum (SBP) and minimum (DBP) BP points are identified for each cardiac cycle using a computer algorithm with the points being visually checked and corrected where necessary. In addition a brachial blood pressure measurement was taken using standard automatic brachial blood pressure monitor.
-  
*Vagal Activity/Autonomic Balance:* Heart rate variability was derived from a three-lead electrocardiograph (ECG). The ECG will be recorded through Meditrace Ag/AgCl spot electrodes. Electrodes were placed on subject’s lower left and lower right rib cage and a third on the right clavicular notch. The right rib cage electrode served as the ground and the remaining two as recording sites. During subsequent analyses R waves were detected using an automated algorithm, allowing IBI to be calculated by the program. The detection of R waves were then visually checked and edited where the automatic detection is incorrect. Power spectrum analysis of the IBI data was conducted to determine autonomic balance.
-  
*Endothelial Dysfunction:* Endothelial functioning was assessed by measuring the hyperemic response to a 5 min occlusion of the brachial artery. Brachial artery occlusion was achieved using a standard blood pressure cuff, while the vascular response to occlusion release was be measured by Endo-PAT 2000 equipment (Itamar, Israel). All procedures were non-invasive.
-  
*Inflammatory markers: S*aliva samples were collected from participant to determine the level of IL-1α, IL-1β, TNFα, IL-6, C-reactive protein, using Bioplex assay kits and the Bioplex instrument. Saliva was collected instead of serum as our previous work has shown that levels of inflammatory proteins correlate well in adolescents and importantly, the sensitivity and detection of cytokines was found to be greater in saliva [88]. Participants collected three 2 mL samples (upon awakening, during the afternoon after school and before going to sleep) each day for two consecutive days at their home, via passive drool. Participants were instructed to avoid eating, drinking, taking medications and brushing their teeth at least 30 min prior to collection. They were also instructed to place samples in their home freezer immediately after collection. Samples were returned on ice and placed in -30 °C freezers until time of assay. All samples were kept frozen from collection to time of processing for bio-assay analysis.

### Sample size requirements and power calculation

A power analysis was conducted prior to commencement of the study in order to provide a guide to sample size requirements. Recruitment was school-based using a cluster sampling scheme to optimize the two-gate screening method being employed (remembering, however, that the RCT is not *cluster-based,* because allocation is at the individual level). Calculations were based on feasible differences in treatment effect at follow-up, where attrition will be greatest. We initially estimated power under assumptions of simple random sampling, and then adjustment was made for the design effect exceeding 1 due to the cluster sampling design.

The treatment effect was estimated to result in group differences of 0.45 SDs for continuous outcome measures and an odds ratio of 2.5 for case level depression, based on preliminary published findings [89]. For α = .05, 120 participants *at follow-up* was estimated to provide 80 % power. Pre-post changes due to intervention in the pilot data were significantly larger than these conservative effect size estimates [48].

Based on our previous experience, we conservatively estimated 10 % attrition from baseline to follow-up, implying that 120/0.90 = 134 adolescents would need to be randomly allocated into the two arms of the study (this was close to the 144 individuals actually randomized). We expected 60 % screening agreement, and 16 % to meet screening cut-off criteria (this was also close to the 27 % who actually met screening criteria). It was conservatively estimated that (i) 70 % meeting screening criteria would also meet diagnostic interview criteria (86 % actually did), and (ii) 50 % meeting diagnostic inclusion would participate in the RCT (65 % actually did). The estimated design effect [90] was 1.44 based on an average cluster-size of 9.275, a coefficient of variation for cluster size of 0.25, and an intra-class correlation of 0.05 on continuous outcome measures. The final estimated required sample size at baseline adjusted for design effect and attrition was 1.44 × 134 = 194 adolescents, which corresponded to estimating need to screen at 194/9.275 = 21 schools. The final actual number of schools screened was 23.

#### Statistical analysis

We plan to examine treatment group differences and differential group changes from baseline to follow-up for outcome measures in all 3 hypotheses using multilevel modeling [91, 92] to account for any cluster sampling effects. Hypothesized mediating effects of sleep improvement on the relationship between treatment condition and outcomes will be assessed using bootstrap confidence intervals [93]. Any recruitment bias between consenters and refusers *after* diagnostic screening, and any differential attrition effects by comparing baseline characteristics of drop-outs and continuing participants, will be investigated using these models.

## Discussion

There is great interest in the possibility that sleep is a modifiable risk factor for the emergence of depression in adolescence. This unique study will provide critical information regarding the effectiveness of a brief sleep intervention for preventing depression, improving wellbeing and enhancing cardiac health in adolescents with anxiety and sleep problems. Given the high prevalence of adolescent depressive disorders, as well as the significant morbidity and mortality associated with both depressive and cardiac disease throughout the lifespan, the implications of an effective intervention of this type for clinical practice and public policy are potentially significant. Indeed, if the intervention proves to be effective it can easily be disseminated to a wide range of clinical settings in primary care, mental health, adolescent health and sleep medicine. The intervention lends itself to flexible modes of delivery (e.g., non-specialist practitioners, group settings, school based, internet and other e-health modes of delivery), further enhancing its translational potential.

### Trial status

At submission of this article, Phases 1–4 had been completed, such that 118 eligible participants in nine parallel groups have completed the interventions and pre/post assessments. One additional participant completed the SENSE Study control group because of a scoring error at the screening phase, but did not meet eligibility criteria, so will be excluded from future analyses. Two-year follow-up assessments began in June 2015 and will be completed by December 2016, as such, the main outcomes of the study (preventative effects) are yet to be assessed.

## Additional files

Additional file 1:
**WHO Trial Registration Data Set_SENSE Study.** (DOCX 72 kb) Additional file 2:
**SPIRIT_Checklist_SENSE Study.** (DOC 123 kb)
