# Supplementary material for: The SENSE Study (Sleep and Education: learning New Skills Early): a community cognitive-behavioural therapy and mindfulness-based sleep intervention to prevent depression and improve cardiac health in adolescence
Source: BMC Psychol. 2015 Nov 4;3:39. doi: 10.1186/s40359-015-0096-x (PMC4634734; doi:10.1186/s40359-015-0096-x)
Supplement: Additional file 1: — WHO Trial Registration Data Set_SENSE Study. (DOCX 72 kb) [file 40359_2015_96_MOESM1_ESM.docx]

**WHO Trial Registration Data Set – The SENSE Study**

| **DATA CATEGORY** | **INFORMATIOn** |
| --- | --- |
| Primary registry and trial identifying number | Australian New Zealand Clinical Trials Registry ACTRN12612001177842 |
| Date of registration in primary registry | 06 Nov, 2012 |
| Secondary identifying numbers | NA |
| Source(s) of monetary or material support | Australian National Health and Medical Research Council Grant (APP1027076) |
| Primary sponsor | Australian National Health and Medical Research Council Grant (APP1027076) |
| Secondary sponsor(s) | NA |
| Contact for public queries | Nicholas B. Allen  Department of Psychology  University of Oregon  Eugene OR 97403-1227  Email: [nallen3@uoregon.edu](mailto:joannamw@unimelb.edu.au)  Telephone: +1 541 346 4075 |
| Contact for scientific queries | Nicholas B. Allen  Department of Psychology  University of Oregon  Eugene OR 97403-1227  Email: [nallen3@uoregon.edu](mailto:joannamw@unimelb.edu.au)  Telephone: +1 541 346 4075 |
| Public title | The SENSE Study (Sleep and Education: learning New Skills Early) |
| Scientific title | The SENSE Study (Sleep and Education: learning New Skills Early): A community cognitive-behavioural therapy and mindfulness-based sleep intervention to prevent depression and improve cardiac health in adolescence |
| Countries of recruitment | Australia |
| Health condition(s) or problem(s) studied | Presence of sleep and anxiety disturbance; prevention of Major Depressive Disorder and improvement in cardiovascular health |
| Intervention(s) | Treatment: Sleep SENSE (a multi-component cognitive-behavioral therapy and mindfulness-based group sleep intervention; incorporating sleep hygiene, stimulus control, cognitive therapy, and mindfulness-based therapy. It involves 7 weekly 90-minute group sessions supported by a range of psycho-educational materials and at-home tasks)  Active Control: Study SENSE (a multicomponent group study skills intervention, incorporating persuasive writing, referencing, note taking and public speaking, administered in the same format and for the same duration as the sleep intervention) |
| Key inclusion and exclusion criteria | Ages eligible for study: 12-17 years Sexes eligible for study: both Accepts healthy volunteers: only accepts at-risk adolescents Inclusion criteria: Early-mid adolescent (12-17 years) identified as being at-risk for depression, defined as reporting high levels of anxiety (Spence Children’s Anxiety Scale ([SCAS], Total Score > 32 and > 38 for males and females respectively) and sleep problems (Pittsburgh Sleep Quality Index ([PSQI], Global Score ≥ 5) on an in school screening. Exclusion criteria: No history of depression, defined as no past or current diagnosis of Major Depressive Disorder, as assessed by a semi-structured clinical diagnostic interview (the Kiddie Schedule for Affective Disorders and Schizophrenia for School-Age Children-Present and Lifetime Version) |
| Study type | Interventional Allocation: randomized (stratified by age, gender, anxiety disorder) Intervention model: parallel assignment Masking: single blind (assessors blinded) Primary purpose: prevention |
| Date of first enrolment | February 2013 |
| Target sample size | 134 |
| Recruitment status | Phase 1-4 complete (screening – post-intervention)  Phase 5 (2-year follow up) began in June 2015 and will be run until December 2016 |
| Primary outcome(s) | 1. The sleep intervention (Sleep SENSE) will improve both subjective and objective indices of sleep quality in a sample of at risk adolescents, and this improvement will persist at a two-year follow-up 2. The sleep intervention will decrease reports of anxiety and mood symptoms immediately after the intervention and prevent the onset of case-level depression over a two-year follow-up period 3. The sleep intervention will improve indices of cardiovascular health at two-year follow-up. In particular, levels of IL-1α, IL-1β, TNFα, IL-6, and C-reactive protein will decrease and endothelial function will improve 4. Benefits to both mental and cardiovascular health will be mediated by the measured improvements in sleep that result from the sleep intervention |
| Key secondary outcomes | 1. The sleep intervention will improve sleep-related behaviours, including bed-time/rise-time variability, bed-time and napping 2. The sleep intervention will improve sleep-related cognitions, including beliefs and attitudes about sleep and sleep knowledge 3. The sleep intervention will decrease reports of pre-sleep hyperarousal, including pre-sleep cognitive arousal pre-sleep somatic arousal 4. The sleep intervention will decrease reports of maladaptive global cognitions, including worry, rumination and low self-efficacy. |
